# The emergence of international comparisons of health inequalities as reflected in the *Scandinavian Journal of Public Health* during its five decades

**DOI:** 10.1177/14034948221079061

**Published:** 2022-03-29

**Authors:** Eero Lahelma, Ossi Rahkonen

**Affiliations:** Department of Public Health, University of Helsinki, Finland

**Keywords:** Comparative studies, health inequality, review, socioeconomic

## Abstract

**Aims:**

We examined the development of research articles published in the *Scandinavian Journal of Public Health* and its predecessors *Acta Socio-Medica Scandinavica* and the *Scandinavian Journal of Social Medicine* from 1969 until 2020 to be able to identify the place of international comparisons of socioeconomic inequalities in health in the journal.

**Methods:**

Altogether 3237 research articles were screened to yield 126 comparative research articles. Examining full texts of the comparative articles led to 13 articles reporting comparisons of health inequalities.

**Results:**

The first one came out in 1972, but the rest only after the mid-1990s. The most common socioeconomic indicator was education, but also occupational class and income was used. The most common health indicator was self-rated health. The articles compared Nordic countries with each other, but also with non-Nordic countries. Although the number of comparative studies on health inequalities was relatively small, there were examples of well-designed studies using advanced methodology. We examined only published journal articles over the past five decades, not submitted but rejected papers.

**Conclusions:**

**In the *Scandinavian Journal of Public Health* and its predecessors, comparisons of health inequalities were few and emerged relatively late, that is, during the past two decades.**

## Introduction

As the French sociologist, Émile Durkheim, wrote in the late nineteenth century ‘there is only one way of proving that a phenomenon is the cause of another, and that is comparing different cases’ [1]. Comparisons are ubiquitous across disciplines, but in health research the term ‘comparative research’ typically refers to studies including cross-national data on countries and larger regions within them [2].

Research within social sciences and public health, including social medicine and medical sociology, has adopted the comparative method. This method has, in a way, provided these disciplines a substitute for experiments in natural sciences and trials in biomedicine, and thus contributed to progress in health research [2]. However, it took time before systematic international comparisons in health research emerged on a larger scale. Reasons behind this include access to comparable data sources across countries, such as surveys and population registers, as well as novel methods of analysis.

Internationally, social class differences in mortality have been studied in Britain earlier than elsewhere. Already in the mid-19th century, Edwin Chadwick’s studies found that poorer people were more likely to suffer from illnesses and premature death. Later in the early 20th century, Britain adopted a standardised Registrar General’s social class classification to be used in mortality statistics. This allowed studies on socioeconomic inequalities in mortality, which were more systematic and looked at changes over time [3].

The Black Report [4] commissioned by the British government and published in the early 1980s, noted that by the late 1970s within-country studies on health inequalities, that is, hierarchical differences in illness and premature death between socioeconomic groups, were available, for example, from Britain and the Nordic countries. However, systematic between-country comparisons of health inequalities remained few, were based on mortality inequalities, and used divergent socioeconomic classifications. Previously, in the Nordic countries geographical differences in health and social and healthcare were in the focus of research on health inequalities [5]. This is likely to be due to large and persisting regional differences in living conditions between northern and southern as well as eastern and western parts of countries such as Finland, Norway and Sweden.

The Nordic welfare construct focused on social class differences in living conditions, with health as a key subdomain [6, 7]. This paved the way for the emergence of research on socioeconomic inequalities in health in the Nordic countries. One of the earliest systematic comparative studies on health inequalities was based on the Scandinavian welfare survey data from 1972 covering Denmark, Finland, Norway and Sweden [8]. Health was measured by an index based on chronic illnesses and socioeconomic position by occupational social class. In Finland, both farmers and manual workers showed a high prevalence of illness, with white collar workers and entrepreneurs showing the lowest prevalence. Also in other Scandinavian countries, illness among manual workers was highest, but its level was much lower across the occupational classes as compared to Finland.

Internationally, socioeconomic inequalities in health became gradually visible among scholars as well as the broader audience. A major impetus was given by the Black Report [4]. This report also shows continuity in the British analyses of health inequalities, which used the standard social class classification from 70 years back. It has become commonplace to judge that the Black Report ‘re-found’ health inequalities and confirmed that in Britain as well as elsewhere large health inequalities persisted and possibly widened over time. However, the within-country data from a couple of countries did not yet allow systematic between-country comparisons on the pattern and magnitude of health inequalities.

The increasing interest in the study of health inequalities after the Black Report has been confirmed by a bibliometric analysis covering the period of 1966–2014 [9]. Until the early 1990s, the number of studies remained low, but after that an exponential growth started resulting in almost 50,000 published scientific papers over the study period. Such growth has been visible in many European countries, with Britain, The Netherlands, Sweden and Finland among the leading countries [10]. However, a quantitative and bibliographic picture on international comparative research of health inequalities is still missing.

The *Scandinavian Journal of Public Health*, with its predecessors, has been a key sociomedical publication forum over five decades. Within the scope of the journal, themes related to the Nordic welfare states, such as socioeconomic inequalities in health and services, have been pursued besides a broader international coverage. For example, within-country health inequalities comparing Nordic as well as non-Nordic countries have been published in regular issues as well as supplements and special issues [11–14]. More recently, the journal has adopted even a stronger emphasis on the drivers of health inequalities [15]. However, we do not know the relative emphasis of all between-country comparisons and comparisons of health inequalities in the journal over its five decades.

## Aim

We make an effort to examine international comparative studies on health inequalities published in the *Scandinavian Journal of Public Health* and its predecessors over five decades, reflecting chiefly the Nordic research input. We wish to show the extent of international comparative studies on health inequalities with regard to all comparative studies as well as all research articles published in the journal. This gives some hints on the journal’s publication profile, and enables us to point out what has been done, what has received less attention and what might need more attention in the future coverage of the published articles.

Our main aim was to examine the emergence of international comparative studies on health inequalities. We do this by identifying studies published in the *Scandinavian Journal of Public Health* and its predecessors *Acta Socio-Medica Scandinavica* and the *Scandinavian Journal of Social Medicine* from 1969 until 2020. Our review examines the emergence of comparative studies on health inequalities within the journals’ total publication output. First, we include all published research articles and examine their trend over time. Second, we include all international comparative articles and examine their trend over time. Third, we examine articles reporting international cross-country comparisons on health inequalities, including their international coverage, as well as the socioeconomic indicator and the health indicator used. Based on our analysis we summarise the main features of the identified comparative studies on health inequalities and raise examples from different decades.

## Data sources: selection of articles

**Figure 1.**
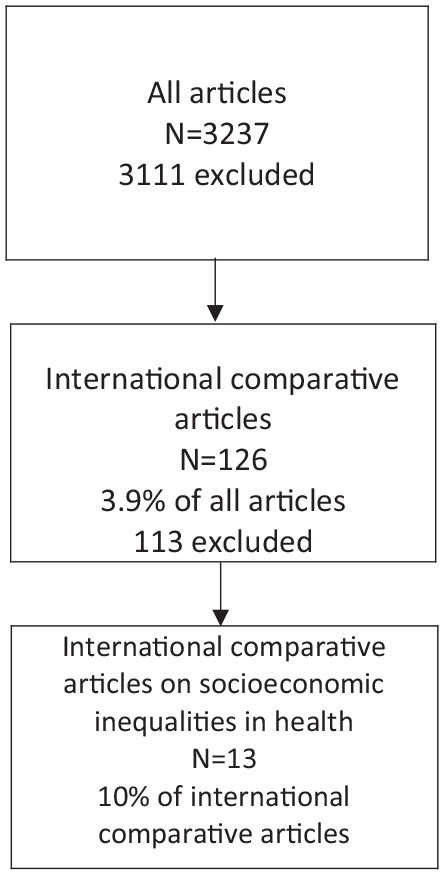
Flowchart of inclusion of research articles published in the *Scandinavian Journal of Public Health* and its predecessors in 1969–2020.

First, our examination covers all volumes of *Acta Socio-Medica Scandinavica* (1969–1972), the *Scandinavian Journal of Social Medicine* (1973–1998) and the *Scandinavian Journal of Public Health* (1999–2020), including regular issues, supplements as well as special issues. The total number of research articles published in 1969–2020 under journal sections ‘article’ and ‘original articles’ as well as ‘reviews’ amounted to 3237 (Figure 1).

Second, both co-authors independently screened all the titles and abstracts of each article in order to identify international comparative studies. Thus, specific search terms to identify articles were not used. We excluded all non-comparative studies, short leading articles presenting issue contents, short comments or letters to the editor without scientific argument, project presentations, book reviews, conference reports as well as editorial news and information. Our screening process yielded 126 articles reporting international comparative studies. Thus, 3111 articles were excluded.

Third, both co-authors screened independently the full texts of comparative articles for identifying our target, that is, international comparative studies on health inequalities covering two or more countries or large regions within two or more countries as units of comparison. Furthermore, we included only studies which used: (a) individual level quantitative data; (b) education, occupational class, income or wealth as the socioeconomic measure; and (c) at least one among the following health indicators: mortality, medically confirmed diseases, self-reported physical or mental ill health, or symptoms of ill health. We excluded aggregate analyses examining recession, austerity or poverty at a country level as well as studies examining non-health outcomes, risk factors or health behaviours, such as body weight, smoking and drinking, which are determinants of health inequalities. There were two disagreements in the inclusion of articles and this led to exclusions after discussion. Thus, 113 articles were excluded. The final number of articles for our review comprised 13 articles, which reported international comparative studies on socioeconomic inequalities in health. We were unable to assess the quality of the studies as our aim was to identify all substantial articles published in the *Scandinavian Journal of Public Health* and its predecessors. However, the quality of each published article was already assessed by reviewers and editors of the journal over its five decades.

## Publication trends and the emergence of comparative studies on health inequalities

**Figure 2.**
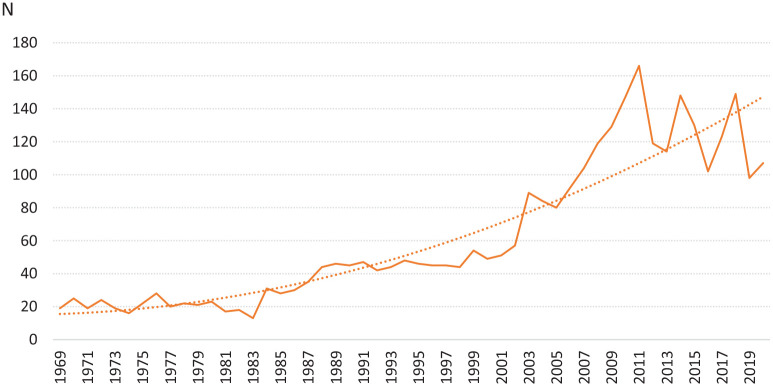
Annual number and polynomial trend of all research articles published in the *Scandinavian Journal of Public Health* and its predecessors in 1969–2020 (*N*=3237).

The total number of research articles over the period of 1969–2020 in the *Scandinavian Journal of Social Medicine* and its predecessors amounted to 3237. Thus, the average number of articles per year is 62. However, the annual number has increased over time, showing a somewhat exponential trend (Figure 2). During the first decade, the number varied around 20, but increased to over 100 since 2007. The steepest growth in the number of articles was during the decade 2000–2010, with some stabilisation over the final decade. This pattern is similar to the growth observed for research articles on socioeconomic inequalities in health over a closely similar period. However, the growth of all articles in the *Scandinavian Journal of Public Health*, with only few socioeconomic comparisons ones, is less steep [9].

**Figure 3.**
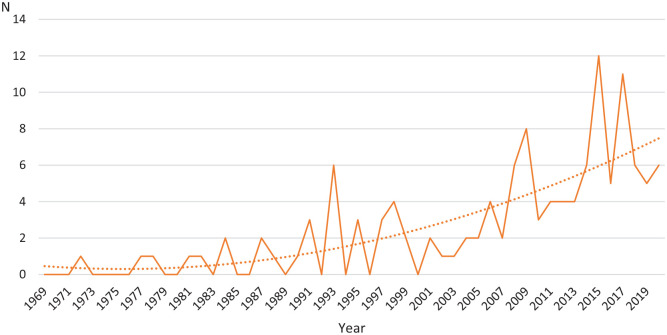
Annual number and polynomial trend of articles reporting international comparisons (*N*=126).

Screening the international comparative research articles for the period of 1969–2020 in the *Scandinavian Journal of Public Health* and its predecessors yielded 126 articles, that is, on average 2.4 comparative articles per year. However, during the two first decades there were only 10 comparative articles and the cumulative number increased after that following the trend of all research articles (Figure 3). As a result, half of all comparative articles fall within the final decade.

## Description of international comparative studies on health inequalities

Among all comparative articles, we found 13 articles reporting studies on systematic international between-country comparisons of socioeconomic inequalities in health. The first one was published in 1972, but the second one no sooner than 1997 (Table I). After that, the speed of publishing increased and during the first decade of 2000 there were four and during the 2010s seven articles reporting on comparisons of health inequalities.

**Table I.** Description of the comparative articles on socioeconomic health inequalities.

Paper	Published year	Countries compared	Data	SEP measure(s)	Health measure(s)
Smedby	1972	Sweden, USA	Nation-wide surveys in Sweden and the US	Education, occupational class, income	Use of medical care
Rahkonen	1997	Finland, Britain	FIN Level of Living Survey UK General Household Survey	Parental and own occupational class	Self-rated health
Berntsson	2001	5 Nordic countries	Children 7–12 years (parental interview)	Family SEP index (education, occupational class, income)	Psychosomatic complaints
Emmelin	2006	Sweden, USA	Population-based cross-sectional health surveys	Education	Self-rated health
Jörgensen	2008	4 Nordic countries	Review	Several SEP measures	Perinatal mortality
Westman	2008	Finland, Sweden	Swedish Level of Living Survey Finnish Health 2000	Education	Self-rated health
Pärna	2010	Estonia, Finland	European Social Survey	Education	Self-rated health
Celeste	2011	Brazil, Sweden	Cross-sectional data	Income	Dental status, use of care
Schütte	2013	31 European countries	European Quality of Life Survey	Education	Self-rated health
Miething	2013	Germany, Sweden	Sweden Level-of-Living Survey German Socio-Economic Panel	Income (education, occupational class)	Self-rated health
Nielsen	2015	5 Nordic countries	Health Behaviour in School- aged Children	Wealth (Family affluence scale)	Psychosomatic symptoms
Lynge	2015	Norway, Denmark	Nordic Occupational Cancer Study (NOCCA)	Occupational class	Colon cancer
Lundetrae	2016	4 Nordic countries	International survey of adult skills (PIAAC)	Education	Self-rated health

SEP: socioeconomic position.

Education was the most frequently used, nine times, indicator of socioeconomic position, whereas occupational class, including parental class, was used six times, and income five times. Four articles included more than one socioeconomic indicator. One article used a family affluence scale, including four items, such as owning a car and the number of bedrooms, and another used composite indices of family socioeconomic position and financial resources.

Most frequently, seven times, studies used self-rated health, also known as self-assessed health or self-perceived health, as an outcome. Inequalities in mortality were examined in one paper only, and the outcome was perinatal mortality. Two studies, both among schoolchildren, examined psychosomatic complaints, and one study examined colon cancer. Two more studies reported inequalities in the use of medical and dental care.

Five Nordic countries, that is, Denmark, Finland, Iceland, Norway and Sweden, were studied in two articles while two others excluded Iceland. One study covered 31 European countries. All articles included at least one Nordic country. Two articles compared Sweden with the US and the rest one Nordic country with Brazil, Britain, Estonia, or Germany (Table I).

## Study examples

Among the 13 comparative studies on health inequalities, some provided just minimal descriptives, whereas some others reported broader and deeper analyses. We picked three illustrative study examples from different decades.

Our first example is a comparative study on socioeconomic inequalities in health by Smedby [16], published as early as 1972 in the *Acta Socio-Medica Scandinavica*. The article was originally presented at the first International Epidemiological Association Nordic meeting held in Malmö, Sweden 1970. The proceedings of the meeting came out as the journal special issue 2–3/1972. The article was based on Smedby’s and his colleagues’ report. They compared Sweden and the US with respect to sociodemographic and socioeconomic determinants of the use of medical and dental care, by using data from nation-wide interview surveys collected in 1964. The study focused on seeing a doctor and a dentist, as well as receiving hospital care. The patterning of inequalities by education, occupational class and family income varied across the type of healthcare. Seeing a doctor was more common among those with higher education and those with higher occupational class. In the US, those with higher family income visited a physician more often than those with lower income, but this was not the case in Sweden. There was a reverse association for hospital care as those in lower positions visited hospital more often. In the US, those with more severe illnesses usually received hospital care regardless of other characteristics and this was even more so in Sweden. Considering the early publication time, this study was well designed and innovative and provided illuminating findings.

The second study example is by Berntsson and colleagues [17] who investigated the association between psychosomatic complaints and their background factors including parental socioeconomic position among 7–12-year-old children from five Nordic countries. Interview survey data were collected in 1996 among a representative sample of parents of Nordic children. Psychosomatic complaints consisted of six items, such as headache, sleeplessness and loss of appetite. Socioeconomic position was measured by parental occupational class, education and disposable family income. Analyses used advanced statistical methods, such as structural equation modelling. In each Nordic country, children from families with low education, manual occupation and low income were most vulnerable in terms of psychosomatic complaints. Some country differences in the socioeconomic differences of complaints were found. The data were collected after the early 1990s’ recession that hit Sweden hard and even harder Finland, where unemployment peaked at 17%. The highest prevalence of psychosomatic complaints was among Finnish children (29%) and the lowest among Swedish children (20%). In Finland, socioeconomic measures were particularly strongly associated with psychosomatic symptoms. The study, however, was cross-sectional and causal associations could not be judged.

The third study example by Miething et al. [18] examined self-rated health and analysed its association with disposable household income in Sweden as well as East Germany and West Germany. A particular interest was in the after-effects of the German reunification. Cross-sectional survey data from Sweden and Germany in 2000 were analysed. The study used advanced methodology, including sensitivity analyses, ordered logistic regression analyses as well as interaction analyses. The authors were aware of difficulties in comparing countries using datasets and measures that are not identical. Focusing on the comparison of income inequalities in health across the three regions, education and occupational class were simultaneously controlled for. There is a risk of overadjustment, but the authors’ aim was to provide a reliable picture of health inequalities and avoid reverse causality. There were income inequalities in health and they varied between the three regional contexts as well as sexes. Analyses of the short and long-term after-effects of the German reunification need to consider the regional variations in health inequalities.

## Discussion

We examined the development of research articles published in the *Scandinavian Journal of Public Health* and its predecessors extending over five decades. Among all published 3237 articles, we identified international cross-country comparisons, and among these, we identified comparisons on socioeconomic inequalities in health. We found, first, altogether 13 comparative articles on health inequalities; second, nearly all of these articles were published after the turn of the millennium; third, the most frequently used health outcome was self-rated health; fourth, the most frequently used socioeconomic indicator was education; and, fifth, all comparisons included at least one Nordic country.

Overall, 13 comparative studies on health inequalities is a relatively small number. Partly, our exclusions contribute to this. Partly, comparative studies on socioeconomic inequalities in health were still rarely published in journals on health research in the 1970s and 1980s. After that comparative studies emerged gradually in Britain, the Nordic countries and elsewhere [19–25]. The reasons behind the emergence of Nordic studies include, for example, interest in the welfare states and the related availability of level of living surveys coordinated between countries. In addition, there are broadly similar national population and health registers, which provide reliable and comprehensive data for Nordic comparisons. The number of studies continued to grow towards the turn of the millennium and after that [26–28]. There are signs of a similar development in the *Scandinavian Journal of Public Health*, in which the strongest growth of comparative articles on health inequalities is seen during 2000–2020. This pattern resembles the growth observed for country-specific research articles on socioeconomic inequalities in health over a largely similar period [9].

Education was the main socioeconomic measure in the comparisons reporting socioeconomic inequalities in health. The measurement of education varied somewhat between countries, and years as well as levels of education were used. Regardless of measurement, education forms an ordinal scale and each person, woman or man, employed or non-employed, has at least some education. In addition, education changes relatively little over the life course, and at least does not decline as income may do. In general, education is a socioeconomic indicator very often used in health research [14].

In the articles reviewed by us, self-rated health was the most often used health indicator, which tells us that many studies were based on survey data. Self-rated health is one of the most common measures in health and welfare research. It reflects generic health and the quality of life in mental as well as physical terms. Questions typically ask about health across scales ranging from excellent to poor health. Self-rated health has been shown to be a reliable and valid measure of general health, and it predicts future healthcare use and mortality [29].

There was almost a complete lack of studies on comparative inequalities in mortality, although an abundance of studies on mortality inequalities have come out in public health and epidemiology journals as well as edited books since Chadwick’s studies in the mid-19th century. Furthermore, the lack of mortality studies was unexpected regarding the availability of Nordic national population registers. This lack may be partly due to the scope of our examination, which excluded aggregate and within-country studies.

Our study has some limitations and a number of factors have likely shaped the picture on the comparisons of health inequalities reported in our review. First, we could include only published papers, but not submitted and rejected papers. Second, many relevant studies, for example, on mortality, have come out as reports and book chapters, which we could not cover. Third, we excluded determinants of health inequalities, such as risk factors and health behaviours. Fourth, our study on one single journal lacks comparisons to other journals on health research. As the number of journals has increased, there have been opportunities to publish comparative studies on health inequalities in journals other than the *Scandinavian Journal of Public Health*. It would be of interest to examine whether the trends are similar or dissimilar in the other journals.

## Conclusions

In general, international comparisons of socioeconomic inequalities in health have increased since the 1990s and the findings of our review reflect this development. A major contribution to comparative research on health inequalities has been made by a series of European and even broader systematic comparisons of health inequalities since the 1980s [10]. Strong comparative research initiatives outside the Nordic countries help understand the relatively small number of comparative studies on health inequalities in the *Scandinavian Journal of Public Health*. Each comparative article on health inequalities in the journal reviewed by us included at least one Nordic country, thus emphasising the ‘Nordicness’ of the forum.

However, health inequalities are universal, but their patterning, absolute and relative magnitude, as well as changes over time, vary across countries. This underscores the importance of future international cross-country comparisons on health inequalities. One can hope and expect to see a growing trend of comparative studies on health inequalities, covering the Nordic as well as non-Nordic countries, to continue in the *Scandinavian Journal of Public Health*.

Health and welfare policies to tackle health inequalities also vary between countries, but so far reducing health inequalities has turned out to be a tough nut to crack. Countries are historically, culturally and economically different, but they can learn from each other how to pursue more efficient egalitarian health and welfare policies. The author of the earliest article in our review, Björn Smedby [16], pointed this out already in 1972. We think that his statement is still valid.
